# Neural Differentiation is Moderated by Age in Scene-Selective, But Not Face-Selective, Cortical Regions

**DOI:** 10.1523/ENEURO.0142-20.2020

**Published:** 2020-05-21

**Authors:** Sabina Srokova, Paul F. Hill, Joshua D. Koen, Danielle R. King, Michael D. Rugg

**Affiliations:** 1 Center for Vital Longevity, University of Texas at Dallas, Dallas, TX 75235; 2 School of Behavioral and Brain Sciences, University of Texas at Dallas, Richardson, TX 75080; 3 Department of Psychology, University of Notre Dame, Notre Dame, IN 46556; 4 School of Psychology, University of East Anglia, Norwich NR4 7TJ, United Kingdom

**Keywords:** cognitive aging, neural differentiation, pattern similarity

## Abstract

The aging brain is characterized by neural dedifferentiation, an apparent decrease in the functional selectivity of category-selective cortical regions. Age-related reductions in neural differentiation have been proposed to play a causal role in cognitive aging. Recent findings suggest, however, that age-related dedifferentiation is not equally evident for all stimulus categories and, additionally, that the relationship between neural differentiation and cognitive performance is not moderated by age. In light of these findings, in the present experiment, younger and older human adults (males and females) underwent fMRI as they studied words paired with images of scenes or faces before a subsequent memory task. Neural selectivity was measured in two scene-selective (parahippocampal place area (PPA) and retrosplenial cortex (RSC)] and two face-selective [fusiform face area (FFA) and occipital face area (OFA)] regions using both a univariate differentiation index and multivoxel pattern similarity analysis. Both methods provided highly convergent results, which revealed evidence of age-related reductions in neural dedifferentiation in scene-selective but not face-selective cortical regions. Additionally, neural differentiation in the PPA demonstrated a positive, age-invariant relationship with subsequent source memory performance (recall of the image category paired with each recognized test word). These findings extend prior findings suggesting that age-related neural dedifferentiation is not a ubiquitous phenomenon, and that the specificity of neural responses to scenes is predictive of subsequent memory performance independently of age.

## Significance Statement

Increasing age is associated with reduced neural specificity in cortical regions that are selectively responsive to a given perceptual stimulus category (age-related neural dedifferentiation), a phenomenon which has been proposed to contribute to cognitive aging. Recent findings reveal that age-related neural dedifferentiation is not always present for all types of visual stimulus categories, and the factors which determine when the phenomenon arises remain unclear. Here, we demonstrate that scene-selective but not face-selective cortical regions exhibit age-related neural dedifferentiation during an attentionally-demanding task. Additionally, we report that higher neural selectivity in the scene-selective parahippocampal place area (PPA) is associated with better memory performance after controlling for variance associated with age group, adding to evidence that neural differentiation impacts cognition across the adult lifespan.

## Introduction

Increasing age has been reported to be associated with reduced specificity and distinctiveness of neural representations, a phenomenon known as age-related neural dedifferentiation (for review, see Koen and Rugg, 2019; Koen et al., 2020). Computational models of cognitive aging suggest that neural dedifferentiation plays a role in age-related cognitive decline (Li et al., 2001; Li and Rieckmann, 2014). Specifically, the phenomenon has been proposed to arise from age-related reductions in neuromodulation, compromising the fidelity of neural representations (see Abdulrahman et al., 2017).

In an early fMRI study of age-related neural dedifferentiation, Park et al. (2004) reported that older adults demonstrated lower neural selectivity in voxels selective for four perceptual categories (houses, chairs, pseudowords, and faces). Although subsequent studies have reported convergent findings, the data suggest that age-related dedifferentiation is not ubiquitous. For example, whereas dedifferentiation is frequently reported in scene-selective (Voss et al., 2008; Carp et al., 2011; Zheng et al., 2018; Koen et al., 2019) and face-selective cortical regions (Park et al., 2004, 2012; Voss et al., 2008; Zebrowitz et al., 2016), null findings for both of these stimulus classes have also been reported (for scenes, Berron et al., 2018; for faces, Payer et al., 2006). The evidence is also divergent for object and word stimuli. Although Park et al. (2004) reported age-related dedifferentiation for objects and orthographic stimuli, subsequent studies have found null age effects for both stimulus classes (objects, Chee et al., 2006; Zebrowitz et al., 2016; Zheng et al., 2018; Koen et al., 2019; words, Voss et al., 2008; see also Wang et al., 2016; Abdulrahman et al., 2017).

Numerous factors likely account for these inconsistent reports, and one such factor might be the attentional demands imposed by the experimental task. Whereas prior studies that employed relatively “passive” viewing tasks have typically reported age-related neural dedifferentiation for both faces (Park et al., 2004, 2012; Voss et al., 2008; Zebrowitz et al., 2016) and object stimuli (Park et al., 2004; but see Chee et al., 2006), studies that employed tasks requiring discriminative judgements on the experimental items have tended to report little or no evidence for neural dedifferentiation (faces, Payer et al., 2006; objects, Koen et al., 2019). In line with reports suggesting that neural selectivity in category-selective cortical regions is modulated by selective attention (Gazzaley et al., 2005, 2008; Baldauf and Desimone, 2014), findings of neural dedifferentiation in the context of passive viewing might have been confounded by age differences in attentional deployment. Therefore, here we examined whether the prior findings of Koen et al. (2019) of null age effects of neural differentiation of objects during an active encoding task extended to faces.

Metrics of neural differentiation have been reported to predict both memory performance for the experimental stimuli (Yassa et al., 2011; Berron et al., 2018; Bowman et al., 2019; Koen et al., 2019; Sommer et al., 2019; for related findings, see Du et al., 2016) and measures of performance on psychometric tests tapping “fluid” processing (Park et al., 2010; Koen et al., 2019). The findings are consistent with the possibility that age-related cognitive decline is driven by neural dedifferentiation. Of importance, however, recent findings suggest that the relationship between neural differentiation and cognitive performance is age-invariant (Koen et al., 2019; Koen and Rugg, 2019), that is, the strength of the relationship does not vary with age. Although an age-invariant relationship does not rule out a role for dedifferentiation in mediating age-related cognitive decline, it does suggest that the contribution of neural selectivity to cognitive performance is stable across the lifespan (for further discussion, see Rugg, 2017).

In the present study, participants underwent fMRI while studying word-face and word-scene stimulus pairs before a memory test. Neural differentiation was operationalized by a univariate differentiation index (Voss et al., 2008; Zebrowitz et al., 2016; Koen et al., 2019) and multi-voxel pattern similarity (Zheng et al., 2018; Koen et al., 2019; Sommer et al., 2019, Trelle et al., 2019) in two face-selective [fusiform face area (FFA), occipital face area (OFA)] and two scene-selective [parahippocampal place area (PPA), retrosplenial cortex (RSC)] regions of interest (ROIs). One aim of the current study was to examine whether the null effects of age in neural differentiation of objects (Koen et al., 2019) extend to face stimuli in the context of an attentionally demanding task. Additionally, we aimed to replicate and extend prior findings regarding age-related neural dedifferentiation for scene stimuli, and the relationship between neural differentiation of scenes with subsequent memory performance and measures of fluid processing.

## Materials and Methods

### Ethics statement

The experimental procedures described below were approved by The Institutional Review Boards of the University of Texas Southwestern Medical Center and the University of Texas at Dallas. All participants provided informed consent before taking part in the experiment.

### Participants

Twenty-seven younger and 33 older adult volunteers were recruited from local communities surrounding The University of Texas at Dallas and the greater Dallas metropolitan area, and were compensated $30/h. All volunteers were right-handed, had normal or corrected-to-normal vision, and were fluent English speakers before the age of five. Participants were excluded if they self-reported a history of cardiovascular or neurologic disease, diabetes, substance abuse, use of medication affecting the central nervous system, or showed evidence of cognitive impairment based on their performance on a neuropsychological test battery (see Neuropsychological testing).

Three younger and three older adult participants were excluded from subsequent analyses for the following reasons: voluntary withdrawal from the study (*N* = 2), behavioral performance which resulted in not having enough trials (<10) in a critical memory bin (*N* = 2), technical malfunction of the equipment (*N* = 1), and an incidental MRI finding (*N* = 1). Additionally, six older participants were excluded due to chance source memory performance, according to our pre-determined cutoff score [probability of source recollection (pSR) < 0.1]. The final sample therefore consisted of 24 young (age range: 18–28 years, 15 female) and 24 older adult (age range: 65–75 years, 14 female) participants. Demographic data and neuropsychological test performance are reported in Table 1.

**Table 1:** Demographic data and results of the neuropsychological test battery (mean, SD) for younger and older adults

	Younger adults	Older adults	*p* value
*N*	24	24	
Age	22.42 (3.24)	70.00 (3.46)	
Years of education	15.46 (2.65)	16.71 (2.44)	NS
MMSE	29.25 (0.90)	29.33 (0.70)	NS
CVLT short delay, Free	13.75 (2.00)	11.88 (2.86)	0.012
CVLT short delay, cued	13.83 (2.32)	13.08 (2.15)	NS
CVLT long delay, free	14.13 (2.11)	12.79 (2.62)	NS
CVLT long delay, cued	14.38 (1.93)	13.46 (2.13)	NS
CVLT recognition, hits	15.71 (0.46)	15.25 (1.07)	NS
CVLT recognition, false alarms	0.33 (0.70)	1.67 (1.61)	0.001
Logical memory I	33.00 (4.76)	28.00 (4.11)	<0.001
Logical memory II	32.00 (4.80)	25.83 (5.49)	<0.001
SDMT	62.33 (11.27)	49.29 (7.91)	<0.001
Trails A (s)	20.20 (5.26)	25.11 (6.46)	0.006
Trails B (s)	44.12 (10.18)	62.48 (16.77)	<0.001
Digit span total	19.71 (4.14)	18.79 (3.49)	NS
Category fluency	23.71 (4.91)	22.46 (5.35)	NS
F-A-S	49.17 (12.85)	46.29 (12.75)	NS
WTAR	42.42 (3.46)	44.54 (4.06)	NS
Raven’s	11.04 (0.86)	9.50 (1.89)	0.001
				
Speed factor (RC1)	–1.47 (2.01)	1.56 (1.68)	<0.001
Memory factor (RC2)	1.53 (1.94)	–1.62 (2.42)	<0.001
Crystallized intelligence factor (RC3)	0.30 (1.42)	–0.39 (1.79)	NS
Fluency factor (RC4)	0.20 (1.18)	–0.10 (1.45)	NS

Digit span total corresponds to the sum of forward and backward digit span.

Speed factor bears a negative number with better performance on tasks of processing speed.

NS = not significant.

Several of the participants in the present study had previously participated in one or more studies reported by our laboratory. Specifically, four older adults participated in the event related potential study reported by Koen et al. (2018), one older adult participated in a prior fMRI study reported by Koen et al. (2019), and four older adults took part in an fMRI experiment first reported by de Chastelaine et al. (2015).

### Neuropsychological testing

All participants completed our standard neuropsychological test battery consisting of the Mini-Mental State Examination (MMSE), the California Verbal Learning Test-II (CVLT; Delis et al., 2000), Wechsler Logical Memory Tests 1 and 2 (Wechsler, 2009), The Trail Making tests A and B (Reitan and Wolfson, 1985), the Symbol Digit Modalities Test (SDMT; Smith, 1982), the F-A-S subtest of the Neurosensory Center Comprehensive Evaluation for Aphasia (Spreen and Benton, 1977), the Wechsler Adult Intelligence Scale-Revised subtests of forward and backward digit span (Wechsler, 1981), Category fluency test (Benton, 1968), Raven’s Progressive Matrices (List 1; Raven et al., 2000), and the Wechsler Test of Adult Reading (WTAR; Wechsler, 2001). Potential participants were excluded before the fMRI session if they scored <27 on the MMSE, >1.5 SD below age norms on any standardized memory test, >1.5 SD below age norms on two or more standardized non-memory tests, or if their estimated full-scale IQ was <100.

The neuropsychological test scores were reduced to four components based on the outcome of a principal component analysis applied to a prior large dataset from our laboratory. The dataset comprised scores from younger, middle aged and older adults (total *N* = 154; de Chastelaine et al., 2016). Four principal components with eigenvalues >1, accounting for 64.1% of the variance, were retained and subjected to the Varimax rotation (Kaiser, 1958). The rotated components (RCs) correspond roughly to processing speed (RC1), memory (RC2), crystallized intelligence (RC3), and fluency (RC4). The neuropsychological tests included in the analysis, their corresponding rotated factor weights, and the proportions of variance accounted for by the RCs are presented in Table 2.

**Table 2:** Factor loadings from the PCA, Varimax rotated, based on dataset previously reported by de Chastelaine et al. (2016)

	Speed (RC1)	Memory (RC2)	Crystallized intelligence (RC3)	Fluency (RC4)
CVLT composite	–0.19	0.84	0.08	–0.15
CVLT recognition, hits	–0.20	0.42	0.23	–0.64
CVLT recognition, false alarms	0.21	–0.69	0.26	–0.17
Logical memory composite	0.10	0.67	0.18	0.02
Trails A (s)	0.91	–0.09	–0.05	–0.14
Trails B (s)	0.85	–0.09	–0.28	0.08
SDMT	–0.59	0.40	0.08	0.30
Digit span	–0.16	0.01	0.80	–0.08
Category fluency	–0.34	0.23	0.14	0.63
F-A-S	–0.12	0.06	0.46	0.57
WTAR	–0.12	0.12	0.79	0.21
Raven’s	–0.33	0.48	0.10	0.05
				
Eigenvalue	3.65	1.70	1.28	1.06
Variance explained (before rotation)	0.20	0.14	0.11	0.09
Variance explained (after rotation)	0.19	0.19	0.15	0.11

### Experimental materials and procedure

#### Experimental procedure and materials

Experimental stimuli were presented using Cogent 2000 software (www.vislab.ucl.ac.uk/cogent_2000.php) implemented in MATLAB 2012b (MathWorks). The stimuli were projected onto a translucent screen attached at the rear of the MRI bore and were viewed through a mirror mounted on the scanner head coil. Participants completed two study-test cycles inside the scanner. For each cycle, study and test phases were each split into two scanning sessions, with a 30-s rest period midway through each session. The critical experimental stimuli were distributed across four study and four test sub-lists, with a single sub-list per scanning session. Therefore, participants’ memory for the first two study sub-lists was tested in two memory test sessions before continuing to the second cycle. The critical stimuli comprised 288 concrete nouns, 96 colored images of male and female faces [face stimuli obtained from Minear and Park (2004) database], and 96 colored images of urban and rural scenes. All images of faces and scenes were scaled at 256 × 256 pixels. An additional 68 words and 40 images were used as fillers at the beginning of each scan session and immediately after each break or as practice stimuli. The critical stimuli were interspersed with 96 null trials (white fixation cross) in both the study and test lists (24 trials per sub-list). Stimuli were selected randomly without replacement to create 24 different stimulus sets for yoked younger and older adult pairs. Study and test trials were pseudorandomized such that participants were not presented with more than three consecutive trials belonging to the same image class, or more than two consecutive null trials.

#### Study phase

**Figure 1.**
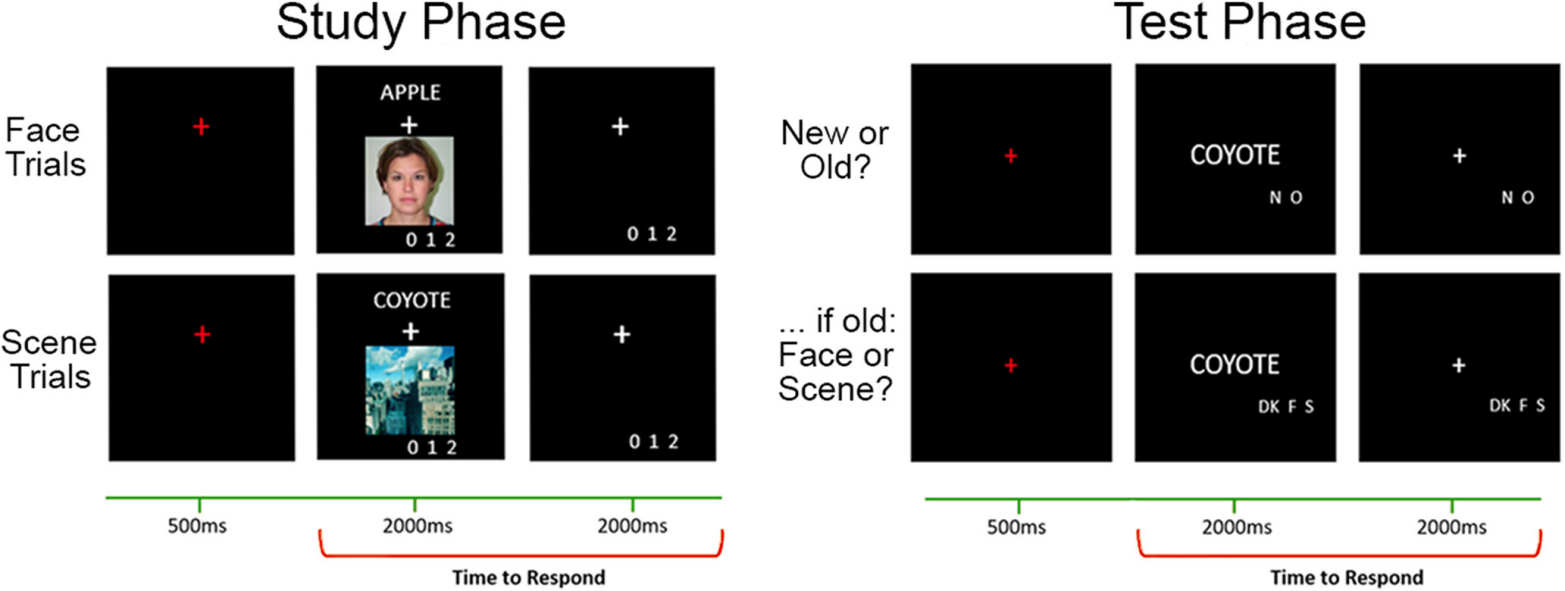
Overview of the encoding task and subsequent memory test. At encoding, participants were asked to “imagine the person interacting with the object denoted by the word” (face trials) or to “imagine the object denoted by the word interacting with the scene” (scene trials).

Participants completed two scanned study-test cycles. Each cycle included two study blocks. The blocks each contained 24 null trials and 48 critical words, half of which were paired with an image of a face and a half paired with a scene image. The word was presented in the upper half of the screen with the image beneath it and a white fixation cross positioned between the two items (see Fig. 1). Words were presented in a white font 30-pt uppercase Helvetica over a black background. A study trial began with a red fixation cross for a duration of 500 ms, followed by the presentation of the word-image pair for 2000 ms. This was followed by a white fixation cross for a further 2000 ms. When a word was paired with a face, the instructions were to imagine the person depicted by the image interacting with the object denoted by the word. For word-scene trials, the task was to imagine a scenario in which the object denoted by the word is interacting with elements of the scene. To ensure adherence to task instructions, participants were asked to rate the vividness of each imagined scenario on a three-point scale: “not vivid,” “somewhat vivid,” to “very vivid.” Responses were recorded with right-hand index, middle and ring fingers using a scanner-compatible button box. Only trials on which ratings were made between 450 and 4500 ms poststimulus onset were included in the analyses described below. Trials attracting multiple responses were excluded from behavioral analyses and included as events of no interest in the fMRI analyses.

**Figure 2.**
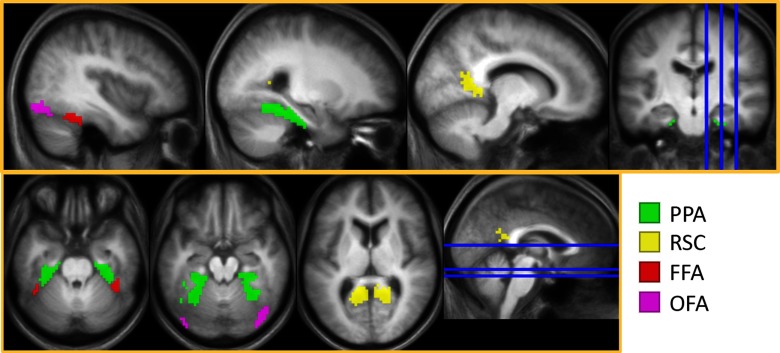
Bilateral scene-selective and face-selective ROIs derived using a second-level GLM contrasting faces and scenes, inclusively masked with Neuromorphometrics in SPM (PPA, FFA, OFA) or with Neurosynth (RSC).

#### Test phase

The test phase was also conducted within the fMRI scanner (the fMRI data will be reported in a separate communication). While undergoing scanning, participants’ memory for the studied items was tested across two test lists (two sub-lists per study-test cycle). Each sub-list comprised 48 studied words, 24 new words, and 24 null trials. Each test trial began with a 500-ms duration red fixation cross, followed by the test word, which was presented for 2000 ms, and a white fixation cross for 2000 ms. Participants were required to indicate whether they remembered studying the test words by making an “Old” or a “New” judgment. Instructions were to respond Old only if they were confident the word had been studied. For test items endorsed Old, participants were prompted to make a source memory judgment, during which they signaled whether the word had been studied along with a face or a scene. An additional “Don’t know” response option was available to discourage guessing. The source memory prompt was presented immediately after the Old/New memory response had been made. Test items receiving a New judgment were followed by a 2000-ms duration white fixation cross. Test responses were made with the index, middle and ring fingers of the right hand on a scanner-compatible button box. The buttons were counterbalanced across participants such that the Old/New judgment were made with the index and middle finger, while the source judgements were counterbalanced across the index, middle, and ring fingers with the constraint that the Don’t know response was never assigned to the middle finger. Analogously to the study phase, trials associated with responses made outside of a 500- to 4500-ms poststimulus window were not considered in the analyses.

### Data acquisition and analysis

#### Experimental design and statistical analysis

The main independent variables in the analyses described below include age group (younger vs older adults), image category of the study trials (faces vs scenes), and the two face-selective and two scene-selective ROIs: FFA and OFA as face-selective ROIs; PPA and RSC as scene-selective ROIs.

Statistical analyses were conducted using R software (R Core Team, 2017) and all tests were considered significant at *p* < 0.05. ANOVAs were performed using the *afex* package (Singmann et al., 2016) and the degrees of freedom were corrected for nonsphericity using the Greenhouse and Geisser (1959) procedure. All *t* tests were performed as Welch’s unequal variance tests using the *t* test function in base R. Effect sizes are reported as partial-η^2^ for the ANOVA results and the package *effsize* (Torchiano, 2019) was used for Cohen’s *d* in pairwise comparisons (Cohen, 1988). Linear regression models were employed using the lm function in base R, and partial correlations were conducted using the function pcor.test in the *ppcor* package (Kim, 2015). Principal components analysis (Hotelling, 1933; Abdi and Williams, 2010) on the neuropsychological test scores was implemented with the *psych* package (Revelle, 2017).

#### Behavioral data analysis

Study and test trials were binned according to their subsequent memory status. We focused on item recognition performance as reflected in the initial Old/New judgment, and source memory performance as indexed by the subsequent “Scene”/“Face”/Don’t know judgment. Trials that received no response or multiple responses were excluded. Item Memory performance was computed as the difference between the overall hit rate and the false alarm rate:

$$Item\;pR=\frac{Item\;Hit}{Old\;Trials}-\frac{False\;Alarms}{New\;Trials}.$$

The hit rate was calculated as the proportion of trials which were correctly endorsed as Old relative to the total number of old trials, regardless of their subsequent source memory judgment. The false alarm rate was calculated as the proportion of new trials incorrectly endorsed as Old relative to all new trials. The overall item recognition accuracy was submitted to a 2 (age group) × 2 (image class) mixed factorial ANOVA.

Additionally, source memory accuracy was computed using a modified single high-threshold model (Snodgrass and Corwin, 1988) according to the following formula (see Gottlieb et al., 2010; Mattson et al., 2014):

$$pSR=\frac{pSource\;Hit-0.5*(1-pSource\;Don^{\prime }t\;Know)}{1-0.5*(1-pSource\;Don^{\prime }t\;know)},$$

where “pSource Hit” refers to the proportion of correctly recognized test items endorsed with a correct source memory judgment at test and “pSource Don’t know” refers to items that were correctly recognized but received a Don’t know source memory response. Given the design of this experiment, our source memory metric necessarily encompasses both face and scene trials. Therefore, we collapsed source memory performance across image type and compared performance between the two age groups with an independent samples *t* test.

Other behavioral measures included reaction time (RT) and vividness ratings for the encoding trials. RT was calculated as the median time to make a vividness rating. Both RTs and the vividness ratings were computed separately for trials corresponding to each image class and binned according to whether or not they were associated with a correct source judgment at test. The vividness ratings and RTs were submitted to separate 2 (age group) × 2 (image class) × 2 (subsequent memory) mixed factorial ANOVAs.

#### MRI data acquisition and preprocessing

fMRI and structural MRI data were acquired at 3T using a Philips Achieva MRI scanner (Philips Medical Systems) equipped with a 32-channel receiver head coil. The functional scans were acquired with a T2*-weighted, blood-oxygen level-dependent echoplanar imaging (EPI) sequence [sensitivity encoding (SENSE) factor = 2, flip angle = 70°, 80 × 78 matrix, field of view (FOV) = 24 cm, repetition time (TR) = 2000 ms, and echo time (TE) = 30 ms]. EPI volumes comprised 34 slices (1 mm interslice gap) at a voxel size of 3 × 3 × 3 mm, acquired in an ascending order and parallel to the anterior-posterior commissure line. Structural images were obtained with a T1-weighted MPRAGE sequence (FOV = 240 × 240, 1 × 1 × 1 mm isotropic voxels, sagittal acquisition).

MRI data were preprocessed and analyzed using a combination of Statistical Parametric Mapping (SPM12, Wellcome Department of Cognitive Neurology, London, United Kingdom) and custom MATLAB scripts. The functional images were realigned to the mean EPI image and slice-time corrected using sinc interpolation to the middle slice. The images were then subjected to reorientation and spatial normalization with respect to a sample-specific template following previously published procedures (de Chastelaine et al., 2011, 2016). Functional images were smoothed with an 8 mm full-width half maximum Gaussian kernel before ROI selection. Estimation of differentiation indices and PSA were conducted on unsmoothed data.

#### MRI data analysis

The analyses reported here focus on the data from the study sessions (analyses of the test data will be reported in a separate paper). The ROIs were derived from univariate fMRI analyses across the four study sessions, which were performed in two stages. In the first stage, separate GLMs were constructed for each participant by sorting the study trials into two categories depending on the trial type: scene trials and face trials. Trials belonging to each of these categories were modeled with a 2 s duration boxcar function onsetting concurrently with the onset of the study word-image pair, convolved with a canonical hemodynamic response function (HRF). Filler trials, null trials, and trials which received multiple or no responses were modeled as covariates of no interest. Additional covariates of no interest included the 30-s duration rest periods midway through each study session and the six regressors representing motion-related variance (three representing rigid-body translation and three for rigid-body rotation along the three axes). Trials with translational displacement >1 mm or with rotational displacement >1° in any direction were modeled as covariates of no interest and hence removed from the analysis. In the second stage, the parameter estimates of the two events of interest were carried over to a second-level random effects 2 × 2 factorial ANOVA with age (younger, older) treated as the between-subjects factor, and trial type (scene, face) as the within-subjects factor.

For the purposes of the differentiation index analyses and the PSA, the unsmoothed data from each of the four total study sessions were concatenated using the *spm_fmri_concatenate* function and subjected to a “least-squares-all” analysis (Rissman et al., 2004; Mumford et al., 2014) to estimate the BOLD response for each trial. Each event was modeled with a 2-s duration boxcar function and convolved with a canonical HRF. The covariates of no interest included the six motion regressors described above and the four session-specific means.

#### ROI selection

Two face-selective (FFA, OFA) and two scene-selective (PPA, RSC) ROIs were empirically defined via a second-level GLM that contrasted scenes and faces (thresholded at *p* < 0.01, uncorrected) across all participants without regard to the factor of age group. The contrasts were inclusively masked with the Neuromorphometrics atlas provided in SPM12. The face > scene contrast was masked with the atlas’s fusiform gyrus and parahippocampal gyrus to derive the FFA mask, and the OFA was defined by inclusively masking the contrasts with inferior occipital and occipital fusiform gyri. The scene > face contrast was masked with the fusiform and parahippocampal gyri to identify the PPA. As Neuromorphometrics does not provide a mask for the RSC, we searched the Neurosynth database using the term “retrosplenial” [search in August 2019, search results false discovery rate (FDR)-corrected at *p* < 0.00,001; Yarkoni et al., 2011] and used the outcome to create the RSC mask. All ROIs were collapsed across the two hemispheres (see Figure 2 and Table 3).

**Table 3:** The voxel size and peak MNI coordinates for each ROI

	Number of voxels	Peak MNI coordinates		
		*x*	*y*	*z*
R. OFA	98	45	–79	–16
L. OFA	24	–45	–85	–10
R. FFA	34	45	–43	–28
L. FFA	10	–42	–49	–25
R. PPA	219	30	–40	–19
L. PPA	249	–27	–46	–16
R. RSC	168	18	–58	14
L. RSC	211	–15	–61	11

L = Left; R = Right.

#### Differentiation index

We computed a differentiation index for each ROI as a measure of the selectivity of neural responses at the regional level (Voss et al., 2008; Zebrowitz et al., 2016; Koen et al., 2019). The differentiation index for a given ROI was computed as the difference between the mean BOLD response for trials of a preferred stimulus class and the mean BOLD response for trials of the non-preferred class, divided by pooled SD:

$$Differentiation\;Index=\frac{\mu_{pref}-\mu_{non\;pref}}{\sqrt{\frac{\sigma_{pref}^{2}+\sigma_{non\;pref}^{2}}{2}}}.$$

Therefore, a higher differentiation index indicates greater selectivity for a given ROI (note that because of the scaling function, the differentiation index is insensitive to individual or group differences in the gain of the HRF mediating between neural activity and the fMRI BOLD response). We computed a differentiation index for each of the four ROIs for each participant. The resulting indices were subjected to a 2 (age group) × 4 (ROI) mixed factorial ANOVA. We conducted an additional ANOVA of the differentiation indices computed only from the trials that went on to receive a source correct memory response. The goal of this additional analysis was to ascertain whether any age differences arising from the original analysis were a reflection of the differential mixing of trial types as a function of age group (on average, young participants had a higher proportion of source correct study trials than did older adults).

Neural dedifferentiation may manifest as a reduced neuronal response to a preferred stimulus category (i.e., neural attenuation), as an elevated response to a non-preferred category (i.e., neural broadening), or as the combination of both phenomena (Park et al., 2012; Koen and Rugg, 2019). The differentiation index is insensitive to this distinction. Thus, we also examined the *β*-parameters, averaged across all voxels within each ROI. The β-parameters were subjected to a 2 (age group) × 2 (ROI) × 2 (image class) mixed factorial ANOVA, separately for face-selective and scene-selective regions.

Finally, to examine whether neural differentiation predicted memory performance or psychometric factor of fluency, for each ROI, we constructed regression models that employed differentiation index and age-group as predictor variables, and, in parallel models, either source or item memory performance as the dependent variable. Initial versions of the models also included the interaction between differentiation index and age group as an additional predictor variable. In no case did the interaction term account for a significant fraction of the variance in performance (*p* > 0.116). Results are reported below for the reduced models that excluded the interaction term.

#### Multivoxel PSA

Multivoxel PSA was conducted in a similar fashion to Koen et al. (2019) to complement the univariate analyses described above. The similarity measures were derived from single-trial, voxel-wise β-parameters (see above, MRI Data analysis). For each participant and ROI, we first computed a within-category similarity metric. This was achieved by computing the correlations across voxels between each study trial and all other study trials belonging to the same image category, subjecting the resulting correlations to a Fisher’s *z* transformation, and averaging them. The between-category similarity was calculated in an analogous fashion except that the correlations were estimated between rather than within image category. The between-similarity and within-similarity was always computed across trials of different scanning sessions to avoid potential bias arising from carry-over effects (Mumford et al., 2014). The similarity index was then computed as the difference between the within-category and between-category similarity metrics. This index can be used as a metric of neural differentiation as it reflects the extent to which different perceptual categories evoke consistent patterns of neural responses within a given ROI. As in the case of the differentiation index described above, this correlation-based metric is insensitive to individual differences in hemodynamic gain.

The similarity indices were subjected to a 2 (age group) × 4 (ROI) mixed factorial ANOVA. As with the analyses of the differentiation indices, we also computed pattern similarity separately for trials that went on to receive a source correct memory response. Additionally, similarity indices were employed in regression analyses aimed at predicting behavioral performance. These analyses were exactly analogous to those conducted on the differentiation indices.

## Results

Demographic data and the outcomes of the neuropsychological test battery are presented in Table 1. The groups were well matched for years of education and MMSE but showed a typical pattern of age-related differences in cognitive performance. Thus, relative to the older group, younger adults had better performance on a subset of declarative memory tests, including the CVLT short free recall test and the logical memory subtests of the WMS. The younger adults also made significantly fewer recognition false alarms on the CVLT recognition memory test and outperformed the older group on the speeded tests (trails A, trails B, and symbol digit modalities) and Raven’s progressive matrices.

The rotated factor loadings (see Materials and Methods) were applied to each participant’s neuropsychological test scores, and the resulting factor scores for the four RCs are presented at the bottom of Table 1. Consistent with the individual neuropsychological tests, there were age differences in the speed and the memory constructs. There were no age differences in the crystallized intelligence or fluency factors.

### Behavioral results

#### Study performance

Mean study RTs and vividness ratings are reported in Table 4, separated by image category and age group. A 2 (age group) × 2 (image category) × 2 (memory: source correct vs source incorrect/don’t know and item misses) mixed factorial ANOVA on the RT data revealed a significant main effect of category, reflecting faster responses in face trials (*F*_(1,46)_ = 5.350, *p* = 0.025, partial-η^2^ = 0.101), but the remaining main effects and all interactions were not significant (*p*s > 0.100). A 2 (age group) × 2 (image category) × 2 (memory) ANOVA on the mean vividness ratings revealed a significant main effect of memory (trials rated as more vivid were associated with better source memory performance; *F*_(1,46)_ = 53.436, *p* < 0.001, partial-η^2^ = 0.537). There was no effect of age (*F*_(1,46)_ = 3.120, *p* = 0.084, partial-η^2^ = 0.064), category (*F*_(1,46)_ = 0.656, *p* = 0.409, partial-η^2^ = 0.015), and no interaction effects (*p*s > 0.180).

**Table 4:** Mean (SD) study phase performance in younger and older adult groups

	Young adults		Older adults	
	Faces	Scenes	Faces	Scenes
Vividness ratings				
Source correct memory	2.42 (.32)	2.44 (.32)	2.24 (.39)	2.18 (.43)
Incorrect memory	2.23 (.42)	2.13 (.51)	2.06 (.46)	2.01 (.49)
				
RT (ms)				
Source correct memory	2369 (678)	2398 (628)	2130 (570)	2266 (524)
Incorrect memory	2351 (658)	2350 (633)	2285 (605)	2327 (579)

#### Memory performance

Memory performance on the experimental task is summarized in Table 5. A 2 (age group) × 2 (image category) mixed factorial ANOVA on item recognition identified a significant main effect of image category (*F*_(1,46)_ = 5.443, *p* = 0.024, partial-η^2^ = 0.106), and a main effect of age group (*F*_(1,46)_ = 10.112, *p* = 0.003, partial-η^2^ = 0.180). There was no significant interaction between the two factors (*F*_(1,46)_ = 0.766, *p* = 0.386, partial-η^2^ = 0.016). The main effect of image class reflected higher item memory performance for words paired with faces relative to scenes. Additionally, overall item recognition performance was significantly greater for younger than older adults. An independent samples *t* test on source memory performance (pSR) revealed a significant difference in favor of the younger group (*t*_(45.12)_ = 3.440, *p* = 0.001, *d* = 1.010).

**Table 5:** Mean (SD) item and source memory performance for younger and older adult groups

	Young adults		Older adults	
	Faces	Scenes	Faces	Scenes
Item hit rate	0.82 (0.15)	0.81 (0.15)	0.70 (0.17)	0.66 (0.14)
False alarm rate	0.13 (0.10)	0.13 (0.10)
				
Proportion source correct	0.83 (0.14)	0.79 (0.16)	0.75 (0.13)	0.68 (0.13)
Proportion source incorrect	0.05 (0.04)	0.06 (0.05)	0.14 (0.07)	0.18 (0.10)
Proportion source don’t know	0.12 (0.13)	0.16 (0.13)	0.12 (0.12)	0.14 (0.13)
				
Item memory	0.69 (0.18)	0.67 (0.17)	0.56 (0.14)	0.52 (0.13)
Source memory (pSR)	0.68 (0.18)		0.51 (0.16)	

Item memory computed as the difference between hit and false alarm rates.

Source memory computed using the single high-threshold model described in Materials and Methods, Data acquisition and analysis, Behavioral data analysis.

#### fMRI differentiation index

**Figure 3.**
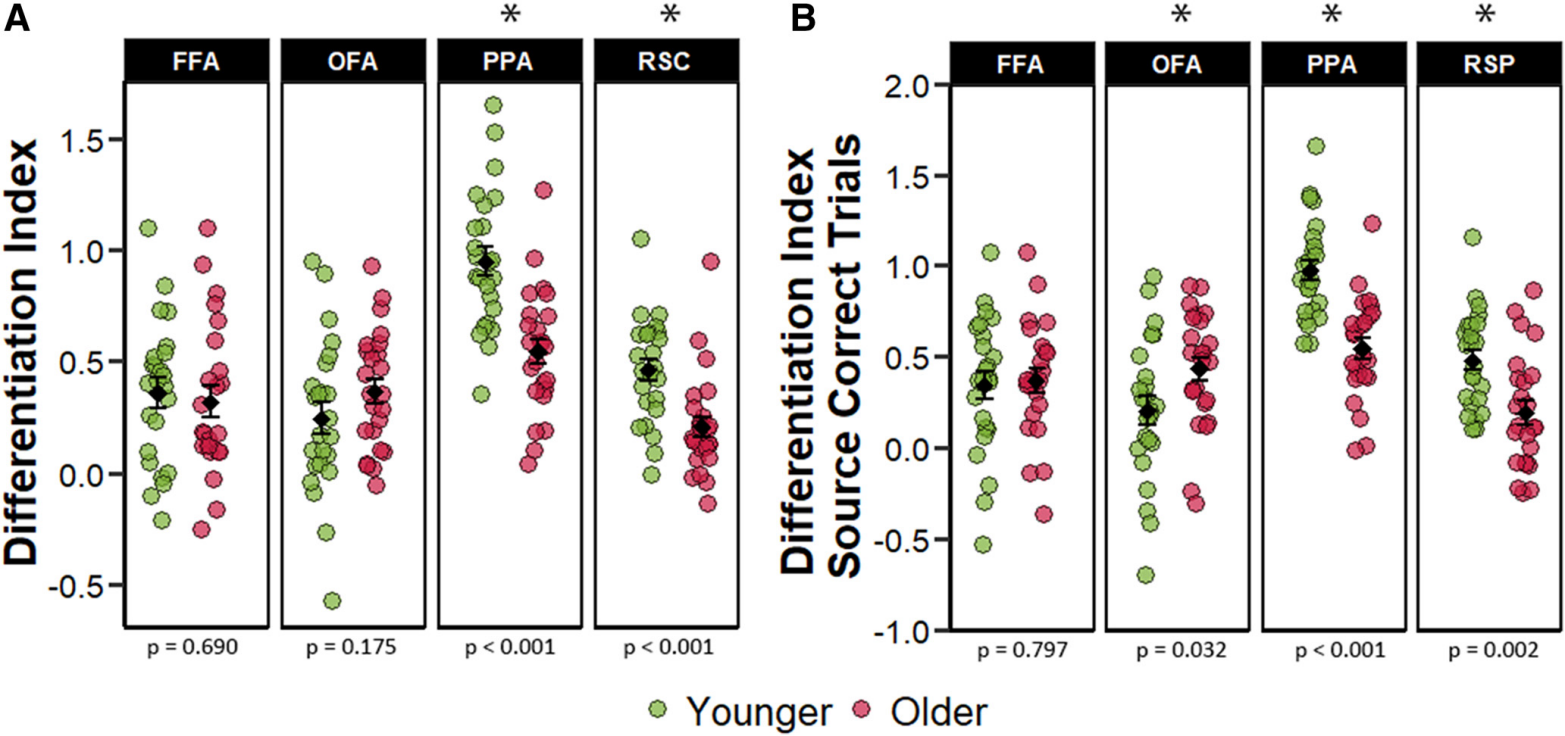
***A***, Univariate differentiation indices collapsed across all trials regardless of subsequent memory performance. ***B***, Differentiation indices computed for only those trials that went on to receive a source-correct response at subsequent retrieval. The error bars around the group means denote ±1 SEM. The *p* values represent the *t* tests comparing younger and older adults in each ROI with an asterisk denoting a statistically significant age difference.

The differentiation indices were subjected to a 2 (age group) × 4 (ROI) mixed factorial ANOVA. The ANOVA revealed a main effect of ROI (*F*_(2.11,96.87)_ = 29.498, *p* < 0.001, partial-η^2^ = 0.391), a main effect of age group (*F*_(1,46)_ = 7.389, *p* = 0.009, partial-η^2^ = 0.138), and a significant age-by-ROI interaction (*F*_(2.11,96.87)_ = 9.025 *p* < 0.001, partial-η^2^ = 0.164). Two follow-up ANOVAs were performed separately for the face-selective and scene-selective ROIs. The 2 (age group) × 2 (scene-selective ROIs) ANOVA resulted in a significant main effect of ROI (*F*_(1,46)_ = 115.71, *p* < 0.001, partial-η^2^ = 0.715), a significant main effect of age group (*F*_(1,46)_ = 24.006, *p* < 0.001, partial-η^2^ = 0.343), and a near-significant age-by-ROI interaction (*F*_(1,46)_ = 3.869, *p* = 0.055, partial-η^2^ = 0.078). As is illustrated in Figure 3*A*,the main effect of age group is driven by reduced neural differentiation in the older age group in both ROIs: PPA (*t*_(45.50)_ = 4.693, *p* < 0.001, *d* = 1.355) and RSC (*t*_(45.95)_ = 3.763, *p* < 0.001, *d* = 1.086). An analogous 2 (age group) × 2 (face-selective ROIs) ANOVA resulted in only a weak trend toward an age-by-ROI interaction (*F*_(1,46)_ = 3.679, *p* = 0.061, partial-η^2^ = 0.074) and no main effect for ROI (*F*_(1,46)_ = 0.637, *p* = 0.429, partial-η^2^ = 0.014), or age group (*F*_(1,46)_ = 0.265, *p* = 0.609, partial-η^2^ = 0.006). Unsurprisingly, therefore, there were null effects of age on neural differentiation in both FFA (*t*_(45.81)_ = 0.401, *p* = 0.690) and OFA (*t*_(42.92)_ = −1.381, *p* = 0.175). Each of the differentiation indices illustrated in Figure 3*A* differed significantly from zero in both age groups (*p*s < 0.002). Together, these results indicate that age group moderated neural differentiation within the scene-selective but not the face-selective ROIs.

In a follow-up analysis, the differentiation index was computed separately for stimulus pairs according to whether they went on to receive a source correct or any form of incorrect response (source incorrect/don’t know and item misses) on the subsequent memory task. A 2 (age group) × 4 (ROI) × 2 (memory status) mixed factorial ANOVA revealed a main effect of ROI (*F*_(2.09,96.21)_ = 23.511, *p* < 0.001, partial-η^2^ = 0.338), a main effect of age group (*F*_(1,46)_ = 6.737, *p* = 0.013, partial-η^2^ = 0.128), a significant age-by-ROI interaction (*F*_(2,09)_ = 6.250, *p* = 0.002, partial-η^2^ = 0.119), and a three-way interaction between age, ROI and memory status (*F*_(1.81,83.16)_ = 4.483, *p* = 0.017, partial-η^2^ = 0.089). However, the analysis did not identify a main effect of memory (*F*_(1,46)_ = 1.714, *p* = 0.197, partial-η^2^ = 0.036), nor a memory-by-age or memory-by-ROI interaction (*F*_(1,46)_ = 2.567, *p* = 0.116, partial-η^2^ = 0.052, and *F*_(1.81,83.16)_ = 0.605, *p* = 0.532, partial-η^2^ = 0.013, respectively). Pairwise follow-up tests failed to identify significant differences between differentiation indices computed separately for the two classes of subsequent memory judgment in any of the ROIs in either age group (*p*s > 0.178).

We went on the examine the differentiation indices only for trials that were later associated with a source-correct memory response to ensure that the age differences reported above were not driven by the differential mixing of source correct and source incorrect trials (given the age differences in source memory; see Materials and Methods). The ANOVA identified a significant main effect of ROI (*F*_(1.89,86.74)_ = 22.401, *p* < 0.001, partial-η^2^ = 0.327), a main effect of age group (*F*_(1,46)_ = 4.890, *p* = 0.032, partial-η^2^ = 0.096), and an age-by-ROI interaction (*F*_(1.89,86.74)_ = 11.103, *p* < 0.001, partial-η^2^ = 0.194). As in the analyses of study trials collapsed across memory performance, we followed up the significant ROI-by-age group interaction with subsidiary 2 (age group) × 2 (face-selective ROIs) and a 2 (age group) × 2 (scene-selective ROIs) ANOVAs. In the scene-selective regions, we identified a significant main effect of age-group (*F*_(1,46)_ = 22.921, *p* < 0.001, partial-η^2^ = 0.333), a main effect of ROI (*F*_(1,46)_ = 133.684, *p* < 0.001, partial-η^2^ = 0.744), but only a trend toward an age-by-ROI interaction (*F*_(1,46)_ = 3.938, *p* = 0.053, partial-η^2^ = 0.079). As evident in Figure 3*B*, the effects of age on neural differentiation within the scene-selective regions were characterized by reduced differentiation indices in both PPA (*t*_(45.98)_ = 5.281, *p* < 0.001, *d* = 1.524), and RSC (*t*_(44.79)_ = 3.359, *p* = 0.002, *d* = 0.970). The analogous analysis in the face-selective regions revealed a significant age-by-ROI interaction (*F*_(1,46)_ = 4.172, *p* = 0.047, partial-η^2^ = 0.083), but the ANOVA did not reveal main effects of age or ROI (*F*_(1,46)_ = 2.013, *p* = 0.163, partial-η^2^ = 0.042 and *F*_(1,46)_ = 0.640, *p* = 0.428, partial-η^2^ = 0.014, respectively). Subsequent pairwise comparisons demonstrated significantly greater differentiation in older relative to younger adults in the OFA (*t*_(43.92)_ = −2.204, *p* = 0.032, *d* = 0.636), but no age differences in the FFA (*t*_(44.94)_ = −0.258, *p* = 0.797, *d* = 0.075). As in the prior analyses, each of the differentiation indices illustrated in Figure 3*B* was significantly different from zero in both age groups (*p*s < 0.019). Overall, restricting analyses to only those encoding trials receiving a subsequent source correct response led to convergent results in scene-selective ROIs, whereby older adults demonstrated lower neural selectivity relative to younger adults.

**Figure 4.**
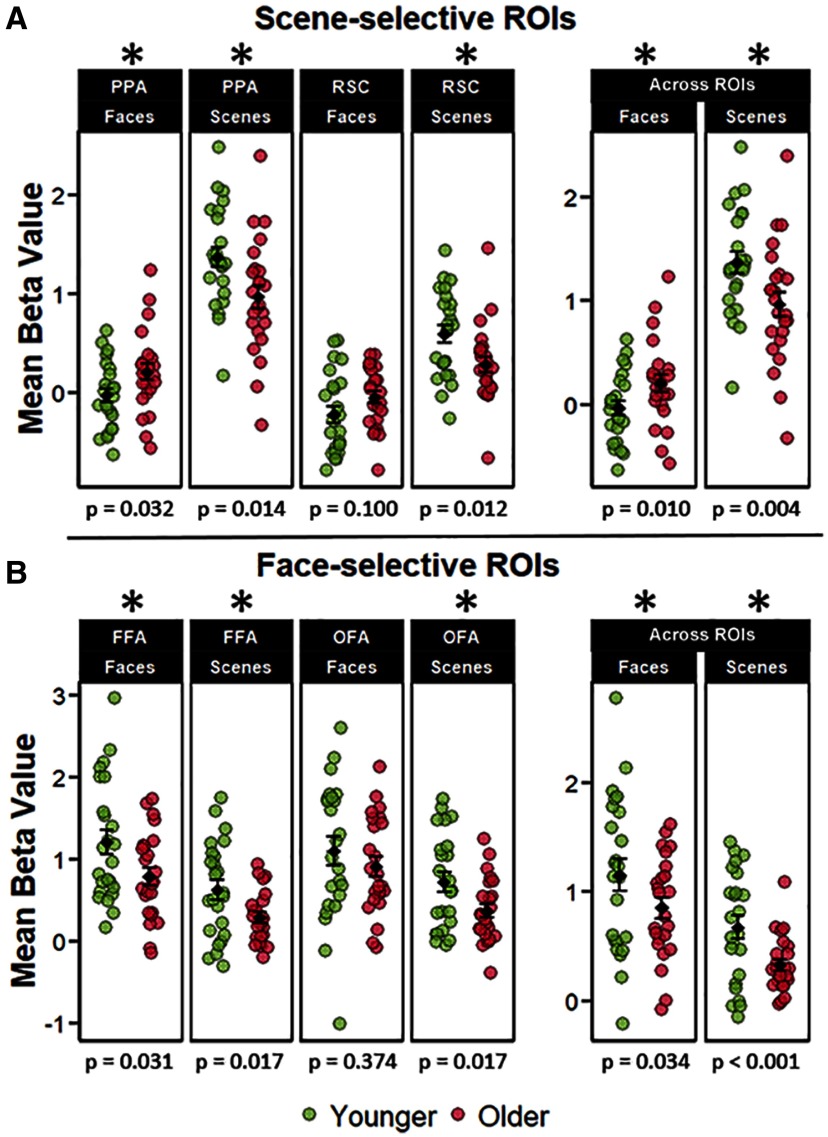
***A***, Across-trial mean β-parameters for face and scene trials in the scene-selective ROIs, including the mean β-parameters collapsed across the scene ROIs. The figure illustrates that age-related neural dedifferentiation in these regions was driven by both broadened responses to faces and attenuated responses to scenes in the older group. ***B***, Across-trial mean β-parameters for face and scene trials in the face-selective ROIs, including the mean β-parameters across the face ROIs. The error bars around the group means denote ±1 SEM. The *p* values represent the *t* tests comparing younger and older adults in each ROI with an asterisk denoting a statistically significant age difference. Unlike in the scene ROIs, parameter estimates were consistently greater for the young relative to the older group.

To further examine age-related dedifferentiation effects in scene-selective regions, we examined whether reduced neural selectivity in older adults resulted from a reduction in BOLD signal for the preferred image category (neural attenuation) or an increase in BOLD signal to the non-preferred category (neural broadening). A 2 (age group) × 2 (scene-selective ROIs) × 2 (image class) mixed factorial ANOVA on the extracted β-parameters revealed a significant main effect of ROI (*F*_(1,46)_ = 125.677, *p* < 0.001, partial-η^2^ = 0.732), and a main effect of stimulus category (*F*_(1,46)_ = 223.252, *p* < 0.001, partial-η^2^ = 0.829), but a null effect of age group (*F*_(1,46)_ = 0.591, *p* = 0.445, partial-η^2^ = 0.013), and a null age-by-ROI interaction (*F*_(1,46)_ = 0.032, *p* = 0.859, partial-η^2^ = 0.001). However, the ANOVA revealed a two-way interaction between stimulus category and age group (*F*_(1,46)_ = 25.859, *p* < 0.001, partial-η^2^ = 0.360), and stimulus category and ROI (*F*_(1,46)_ = 65.59, *p* < 0.001, partial-η^2^ = 0.588). The three-way interaction was not significant (*F*_(1,46)_ = 1.553, *p* = 0.219, partial-η^2^ = 0.033). As is evident from Figure 4*A*, there was an attenuated BOLD response to scenes in older participants across both scene ROIs (*t*_(44.94)_ = −2.894, *p* = 0.005, *d* = −0.591), accompanied by an elevated response to face stimuli (*t*_(44.94)_ = 2.659, *p* = 0.009, *d* = 0.543). Thus, age-related neural dedifferentiation in the scene-selective ROIs was driven by a combination of attenuated BOLD response to scenes and increased responses to faces.

Although no age differences in neural differentiation were observed in the face-selective ROIs, we performed an analysis analogous to that described in the preceding paragraph. Figure 4*B* illustrates the mean BOLD response to face and scene stimuli in these regions. We employed an analogous 2 (age group) × 2 (ROIs) × 2 (image class) ANOVA on the extracted β-parameters. The ANOVA identified main effects of category (*F*_(1,46)_ = 64.107, *p* < 0.001, partial-η^2^ = 0.582) and age group (*F*_(1,46)_ = 5.775, *p* = 0.020, partial-η^2^ = 0.112) and a null effect of ROI (*F*_(1,46)_ = 0.382, *p* = 0.540, partial-η^2^ = 0.008). Unlike in the analysis reported for the scene-selective ROIs, the ANOVA did not identify a significant interaction between age group and category (*F*_(1,46)_ = 0.132, *p* = 0.711, partial-η^2^ = 0.003), and the interaction between age group and ROI was also not significant (*F*_(1,46)_ = 1.241, *p* = 0.271, partial-η^2^ = 0.026). Lastly, the three-way interaction between age group, category, and ROI also failed to attain significance (*F*_(1,46)_ = 3.016, *p* = 0.089, partial-η^2^ = 0.062). The null effects for the interactions involving the factors of age groups and stimulus category are consistent with the outcome of the analysis of the dedifferentiation indices derived from the face-selective ROIs described previously.

#### PSA

**Figure 5.**
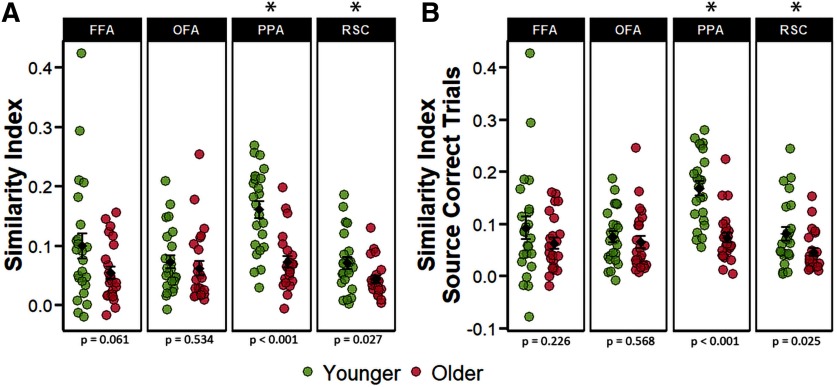
***A***, Within-between similarity indices computed collapsing across memory performance. ***B***, Within-between similarity indices computed for only those trials that went on to receive a source-correct response at subsequent retrieval. The error bars around the group means denote ±1 SEM. The *p* values represent the *t* tests comparing younger and older adults in each ROI with an asterisk denoting a statistically significant age difference.

Multivoxel PSA (Kriegeskorte et al., 2008) was employed as a complement to the analysis of the differentiation index described above. We computed a within-between similarity metric in each ROI as an index of selectivity to the ROI’s preferred relative to the non-preferred stimulus class (see Materials and Methods). Analogous to the analyses of the differentiation index, the initial 2 (age group) × 4 (ROI) mixed factorial ANOVA was employed on the within-between similarity indices computed across all trials regardless of subsequent memory status. This revealed significant main effects of ROI (*F*_(2.35,108.24)_ = 11.924, *p* < 0.001, partial-η^2^ = 0.206), and age group (*F*_(1,46)_ = 12.855, *p* < 0.001, partial-η^2^ = 0.218), along with a significant two-way interaction (*F*_(2.35,108.24)_ = 4.981, *p* = 0.006, partial-η^2^ = 0.098). A subsequent 2 (age group) × 2 (ROI) mixed ANOVA focusing on just the scene-selective ROIs yielded a significant main effect of ROI (*F*_(1,46)_ = 71.020, *p* < 0.001, partial-η^2^ = 0.607), a main effect of age (*F*_(1,46)_ = 20.273, *p* < 0.001, partial-η^2^ = 0.306), and a significant age-by-ROI interaction (*F*_(1,46)_ = 19.077, *p* < 0.001, partial-η^2^ = 0.293). An analogous 2 (age group) × 2 (ROI) ANOVA on the data from the face-selective ROIs failed to identify a significant age-by-ROI interaction (*F*_(1,46)_ = 0.191, *p* = 0.174, partial-η^2^ = 0.040), nor did it reveal significant main effects of ROI (*F*_(1,46)_ = 0.575, *p* = 0.452, partial-η^2^ = 0.012), or age group (*F*_(1,46)_ = 3.091, *p* = 0.085, partial-η^2^ = 0.063). Follow-up pairwise comparisons examining age differences in each of the four ROIs revealed significantly lower similarity metrics for scenes in both the PPA (*t*_(40.50)_ = 5.191, *p* < 0.001, *d* = 1.498) and RSC (*t*_(37.66)_ = 2.290, *p* = 0.027, *d* = 0.660). We did not however detect any age differences in similarity indices for faces within face-selective ROIs: FFA (*t*_(33.06)_ = 1.939, *p* = 0.061, *d* = 0.560) and OFA (*t*_(45.46)_ = 0.626, *p* = 0.534, *d* = 0.181; Fig. 5*A*). The similarity indices differed significantly from zero in all ROIs in both age groups (*p*s < 0.001). These results indicate that, when computed across all encoding trials, within – between pattern similarity was moderated by age in the scene-selective but not the face-selective ROIs.

As with the analyses of the differentiation index, the pattern similarity indices were also computed separately for trials binned into two categories depending on if the trial received a correct source memory response or not at retrieval. A 2 (age group) × 4 (ROI) × 2 (memory status) mixed factorial ANOVA resulted in a main effect of age group (*F*_(1,46)_ = 12.894, *p* < 0.001, partial-η^2^ = 0.219), a main effect of ROI (*F*_(2.34,107.47)_ = 10.873, *p* < 0.001, partial-η^2^ = 0.191), an age-by-ROI interaction (*F*_(2.34,107.47)_ = 4.480, *p* = 0.010, partial-η^2^ = 0.089), and a three-way interaction between age, ROI, and memory status (*F*_(2.39,109.99)_ = 3.542, *p* = 0.025, partial-η^2^ = 0.071). The analysis did not identify a main effect of memory (*F*_(1,46)_ = 3.074, *p* = 0.098, partial-η^2^ = 0.063), nor any two-way interactions between memory and age group or ROI (*p*s > 0.213). Subsequent pairwise comparisons demonstrated that the pattern similarity indices computed separately for the two classes of memory judgment were not significantly different from each other in either ROI in either age group (*p*s > 0.140).

For the reasons described above (see Materials and Methods), we repeated the foregoing analyses using only those trials that went on to give rise to a correct source memory judgment, allowing an assessment of whether age differences in pattern similarity were driven by age differences in the number of successful memory trials contributing to the similarity metrics. A 2 (age group) × 4 (ROI) mixed ANOVA revealed significant main effects of age (*F*_(1,46)_ = 12.071, *p* = 0.001, partial-η^2^ = 0.208) and ROI (*F*_(2.34,107.43)_ = 10.550, *p* < 0.001, partial-η^2^ = 0.187), along with significant age by ROI interaction (*F*_(2.34,107.43)_ = 5.325, *p* = 0.004, partial-η^2^ = 0.104). A follow-up ANOVA on the data for the scene-selective ROIs revealed significant main effects of age group (*F*_(1,46)_ = 20.830, *p* < 0.001, partial-η^2^ = 0.312) and ROI (*F*_(1,46)_ = 58.860, *p* < 0.001, partial-η^2^ = 0.561) as well as an age-by-ROI interaction (*F*_(1,46)_ = 16.221, *p* < 0.001, partial-η^2^ = 0.261). ANOVA of the face-selective ROIs failed to identify any significant effects: age (*F*_(1,46)_ = 1.647, *p* = 0.206, partial-η^2^ = 0.035), ROI (*F*_(1,46)_ = 0.320, *p* = 0.574, partial-η^2^ = 0.007), age-by-ROI interaction (*F*_(1,46)_ = 0.558, *p* = 0.459, partial-η^2^ = 0.012). As Figure 5*B* illustrates, the similarity indices demonstrated age-related reductions in both the PPA and RSC (*t*_(41.62)_ = 5.543, *p* < 0.001, *d* = 1.600, and *t*_(37.12)_ = 2.328, *p* = 0.025, *d* = 0.672, respectively), while age effects were absent in the two face-selective ROIs (*t*_(33.53)_ = 1.230, *p* = 0.226, *d* = 0.356 and *t*_(45.54)_ = 0.575, *p* = 0.568, *d* = 0.166; in the FFA and OFA, respectively). Similarity indices were however significantly different from zero in all ROIs and age groups (*p*s < 0.001). Thus, as with the differentiation index, when PSA was restricted to encoding trials associated with a correct subsequent source memory judgment robust age effects were evident in scene-selective but not face-selective ROIs.

### Relationship between neural differentiation and subsequent memory performance

**Figure 6.**
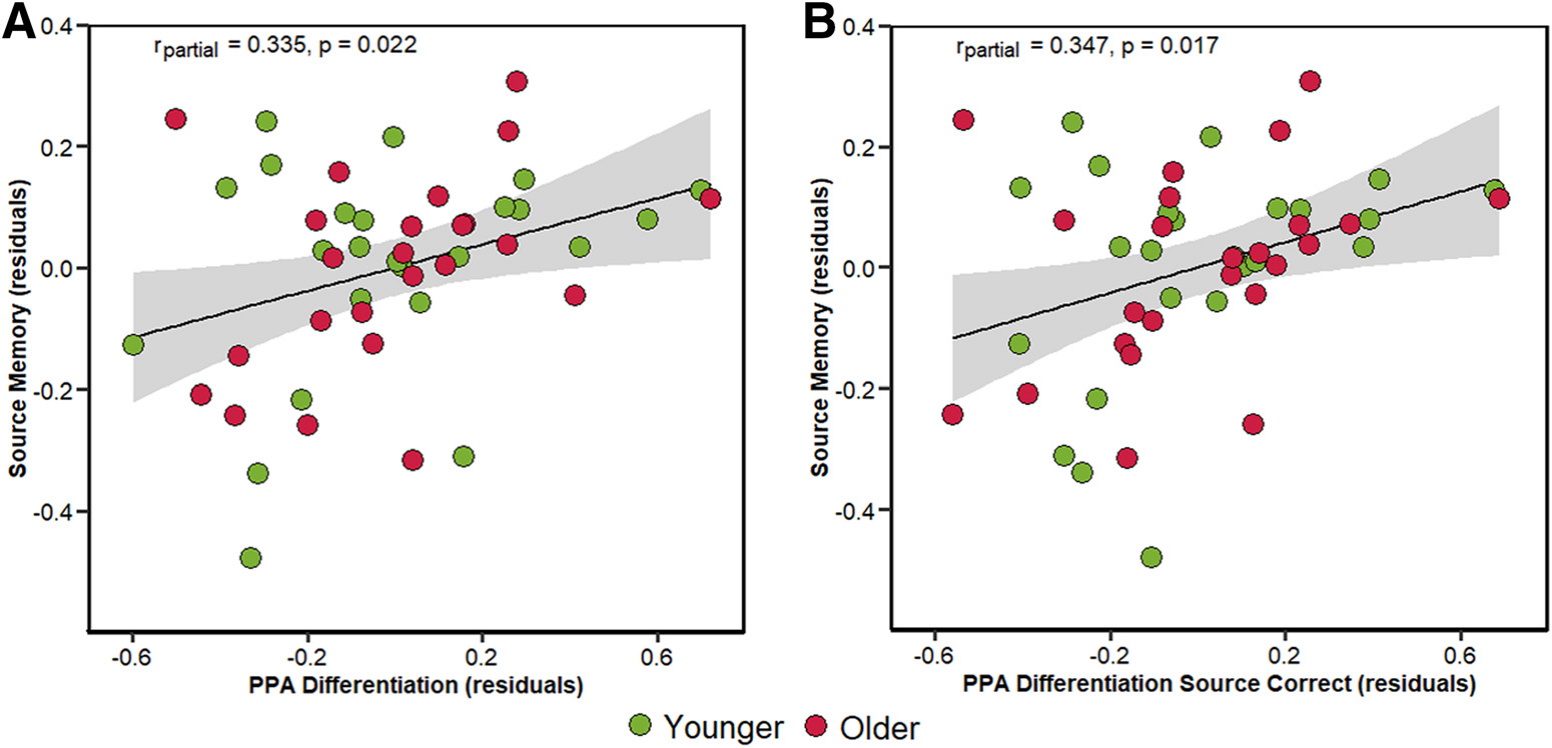
Scatterplots illustrating the partial correlations (controlling for age group) between PPA differentiation indices with source memory performance. Plot ***A*** illustrates the relationship between source memory and differentiation index collapsed across all encoding trials. Plot ***B*** illustrates the same relationship but restricted only to the trials that went on to receive a source correct memory response.

In light of prior findings (Koen et al., 2019), and as described in Materials and Methods, we ran a series of multiple regression analyses in which age group and the differentiation indices from each ROI were employed as predictors of subsequent source and item memory performance. As described in Materials and Methods, the initial multiple regression models included the ROI-by-age interaction terms, however, in no case was the interaction significant (*p* > 0.116). Therefore, Table 6 presents the partial correlations between neural differentiation and performance after controlling for age group. As is evident from the table, the partial correlations between differentiation indices and source memory performance achieved significance only in the PPA. This was the true both when computing the differentiation index collapsing across memory performance and when selecting only the source-correct trials. Moreover, these relationships between differentiation in the PPA and source memory performance remained significant after controlling for both age and item memory performance (collapsed across all trials: *r*_partial_ = 0.334, *p* = 0.023; source-correct trials: *r*_partial_ = 0.314, *p* = 0.033). The partial relationships controlling for age group are illustrated in Figure 6. Analogous analyses were conducted for the pattern similarity indices: no significant relationships between similarity indices and memory performance were identified (*p* > 0.092; data and figures available from corresponding author on request).

**Table 6:** Partial correlations (*p* values) between item memory and source memory performance and differentiation index when controlling for age group

	Collapsed across all trials		Source-correct trials	
	Item memory	Source memory	Item memory	Source memory
FFA	–0.145 (0.330)	–0.117 (0.432)	–0.083 (0.581)	–0.010 (0.945)
OFA	0.071 (0.635)	0.086 (0.567)	0.149 (0.318)	0.08 (0.565)
PPA	0.140 (0.347)	0.335 (0.022)	0.180 (0.225)	0.347 (0.017)
RSC	0.096 (0.519)	0.155 (0.299)	0.037 (0.805)	0.101 (0.498)

The differentiation indices were computed either across all encoding trials (first two columns) or only for those encoding trials that were associated with a source-correct memory response (second two columns).

### Relationship between neural differentiation and neuropsychological test performance

Given prior findings of a positive, age-invariant, relationship between the PPA differentiation index and the fluency component derived from the neuropsychological test battery (see Introduction), we examined whether a similar relationship was evident in the present study. When collapsed across all trials regardless of subsequent memory, the partial correlation (controlling for age) between the differentiation index and fluency factor scores was not significant in either the PPA (*r*_partial_ = −0.009, *p* = 0.951) or the RSC (*r*_partial_ = 0.112, *p* = 0.454). The relationship was also absent when the differentiation index was derived from source correct trials only (PPA: *r*_partial_ = 0.105, *p* = 0.482; RSC: *r*_partial_ = 0.170, *p* = 0.255).

## Discussion

The current study employed a combination of univariate and multi-voxel analyses to examine age effects on category-level neural selectivity (neural differentiation) during the encoding of images of faces and scenes before a subsequent memory test. Neural selectivity was examined in two scene-selective and two face-selective ROIs. The univariate and pattern similarity measures yielded convergent results indicating that scene-selective but not face-selective regions demonstrated reduced category-level selectivity with older age, that is, age-related neural dedifferentiation. The findings add to the already large literature describing age-related neural dedifferentiation effects (for review, see Koen and Rugg, 2019; Koen et al., 2019, 2020), and importantly, also add to evidence suggesting that while the phenomenon is highly robust for scene stimuli, it is more elusive for other stimulus classes: faces in the present case, and objects in Koen et al. (2019). Additionally, analogous to the findings of Koen et al. (2019), the univariate metric of neural differentiation for scenes in the PPA demonstrated a positive, age-invariant, relationship with source memory performance.

Turning first to the behavioral findings, we observed no age differences either in study RT or in the vividness ratings assigned to the study items. Therefore, the age differences we identified in neural differentiation are unlikely to reflect the confounding effects of either of these variables. At test, younger adults outperformed their older counterparts in respect of both item and source memory performance, findings consistent with an extensive prior literature (for review, see Old and Naveh-Benjamin, 2008; Koen and Yonelinas, 2014). Given these age differences in memory performance, we examined neural differentiation indices derived not only from all experimental items (as in prior studies) but also from only those study trials attracting correct source judgments. The results of the two analyses revealed that the findings of age-related neural dedifferentiation in the scene-selective ROIs were not confounded by differential neural activity associated with versus unsuccessful memory encoding.

Age-related reductions in neural specificity have been linked to cognitive declines associated with healthy aging (Koen and Rugg, 2019). This putative link is motivated by the notion that age-related weakening of dopaminergic neuromodulation results in reduced neural signal-to-noise and hence reduced specificity of neural representations (Li et al., 2001; Li and Rieckmann, 2014; see also Abdulrahman et al., 2017). The proposal that age-related neural dedifferentiation plays a role in cognitive decline receives further support from findings that dedifferentiation is associated with lower memory performance (Yassa et al., 2011; Berron et al., 2018; Bowman et al., 2019; Koen et al., 2019) and lower fluid processing ability (Park et al., 2010; Koen et al., 2019). These findings suggest that the neural specificity of perceptual representations plays a role not only in subsequent memory performance but also in broader aspects of neural efficiency and cognition. However, although increasing age is undoubtedly associated with reduced neural selectivity, the existing evidence suggests that the relationship between neural differentiation and cognitive performance is not moderated by age, that is, it is age-invariant (Koen and Rugg, 2019). The present findings of an age-invariant relationship between scene differentiation in the PPA and subsequent source memory performance add to this evidence. These findings serve as a conceptual replication of those reported by Koen et al. (2019), although in that experiment, PPA differentiation was related more strongly to item than to source memory performance. This disparity likely reflects the different experimental procedures: whereas in the present study, category exemplars served as the contextual features targeted in the source memory test, in Koen et al. (2019), the exemplars were the test items themselves.

For reasons that are presently unclear, we failed to replicate the finding (Koen et al., 2019) of a relationship between PPA differentiation and scores on a psychometric fluency factor. Prior studies of neural differentiation have reported a positive relationship between scores on neuropsychological tests tapping fluid intelligence, but not other measures, such as crystallized intelligence (Park et al., 2010; Koen et al., 2019), or the psychometric factors of memory and processing speed (Koen et al., 2019). Although the lack of a significant relationship between differentiation and the fluency component in the present study runs counter to the findings discussed above, we note that the modest effect size for the relationship reported in the study of Koen et al. (2019; *r* = 0.35) constrains the likelihood of replication in studies employing relatively small samples sizes, as was the case here.

As noted in the Introduction, evidence for age-related neural dedifferentiation in the visual domain appears to be most consistent for scenes and faces. Thus, the present findings for scenes in the PPA and RSC are fully consistent with prior findings, whereas the null effects we report for faces in FFA and OFA run counter to several prior results (Park et al., 2004, 2012; Voss et al., 2008; but see Payer et al., 2006). There are several factors that, either jointly or in combination, might account for these disparate findings. One factor concerns the presentation format of the stimuli. Whereas the faces in the present study were rendered in color, as best we can determine, prior studies reporting age-related differentiation for faces all employed gray-scale images. A second factor concerns the processing demands placed on the participants: as we noted in the Introduction, whereas most prior studies reporting age effects on face specificity employed relatively passive viewing conditions (Park et al., 2004, 2010; Voss et al., 2008; Zebrowitz et al., 2016; but see Goh et al., 2010; Burianová et al., 2013), here, we employed a task that required active engagement with the experimental stimuli (as did Payer et al., 2006). If, as has been suggested (see Introduction), older adults have a greater tendency to “zone out” during passive viewing, the resulting reduction in attention to the experimental stimuli may manifest as reduced neural selectivity (for a similar account of inconsistent findings for objects, see Koen et al., 2019). Additionally, whereas prior studies reporting age-related differentiation typically employed blocked experimental designs, here we employed an event-related design in which different category exemplars were presented in an unpredictable order. Lastly, we cannot rule out the possibility that younger and older adults adopted different cognitive strategies when encoding the word-face and word-scene study pairs. Although no age effects were observed for the vividness ratings of these scenarios, it is conceivable that while younger adults allocated attention relatively evenly between the words and images, older adults may have focused less on word – image integration and more on the image itself. Therefore, as neural selectivity of category-selective cortical regions has been reported to be modulated by selective attention (Gazzaley et al., 2005, 2008; Baldauf and Desimone, 2014), age differences in neural differentiation for face stimuli may be blunted if older adults focus more on the elements of the facial features when completing the task. However, heightened attention to elements of the stimuli on the part of older adults is unlikely to explain the phenomenon of reduced neural selectivity observed in scene-selective ROIs.

While some combination of the above-mentioned factors might account for the absence of age-related neural dedifferentiation for faces in the present study, they offer no insight into why dedifferentiation effects for scenes are so robust. Relevant to this question, a recent “lifetime experience hypothesis” (Koen and Rugg, 2019) posits that neural differentiation might be moderated by prior experience that accrues over the lifespan. The hypothesis proposes that accumulating lifetime experience facilitates the assimilation of novel category exemplars into perceptual schemas (Gilboa and Marlatte, 2017). If scene processing becomes increasingly schema-dependent with age, age-related neural dedifferentiation in scene ROIs might reflect more efficient assimilation of scene information into relevant schemas. As was noted by Koen et al. (2019), this proposal receives support from their finding that age-related neural dedifferentiation in the PPA took the form of an age-related reduction in neural responses to scenes (neural attenuation), as was also the case in the present study. By contrast, schemas for some other stimulus categories, such as canonical objects, high frequency words, and, possibly, faces, develop more rapidly and are largely fully formed by adolescence or early adulthood (Germine et al., 2011). By this view, therefore, the present findings of null age effects for face differentiation reflect the fact that young and older adults possess equally well-formed face schemas.

The mixed evidence for age differences in neural selectivity for different perceptual categories might also be explained by age differences in the perceptual processing of complex visual stimuli. For instance, age differences in neural differentiation may be more pronounced when viewing stimuli that comprise combinations of multiple, unpredictable features, such as scenes rather than faces. Notably, it has been reported that PPA activity is strongly modulated by scene complexity (Chai et al., 2010), whereby increasing complexity is associated with greater activity in the region (for review, see Aminoff et al., 2013). If, as has been suggested (Boutet et al., 2019; Meng et al., 2019), older adults are less able to differentiate visual detail, then age differences in neural selectivity in the PPA might be anticipated. In contrast, the null effects of age in neural selectivity for exemplars of canonical objects, words, or human faces, might reflect their relatively low visual complexity, along with, perhaps, higher schema congruency (see above).

We note a number of limitations of the present study. First, measuring neural selectivity at the category level might not provide a sensitive enough measure to detect age differences in the fidelity of face (or object) representations, and it is possible that item-level measures would yield different findings (Goh et al., 2010; St-Laurent et al., 2014; Sommer et al., 2019; Trelle et al., 2019). Second, it is unclear to what extent the present (and previous) findings reflect age differences in the variability or the shape, as opposed to the gain (see Materials and Methods), of stimulus-elicited hemodynamic responses (D'Esposito et al., 2003). Third, like all prior studies of age-related neural dedifferentiation, the present study employed a cross-sectional design. Hence, the reported age differences cannot unambiguously be attributed to the effects of aging as opposed to some correlated confounding factor such as a cohort effect (Rugg, 2017).

In conclusion, although increasing age is associated with reduced neural differentiation between different visual categories, the present study adds to the evidence that this is easier to demonstrate for visual scenes than for other visual categories. In addition, the age-invariant relationship identified here between scene-related neural differentiation and source memory performance adds to prior evidence that neural differentiation is predictive of individual differences in cognitive performance across much of the adult lifespan: lower neural differentiation is associated with lower cognitive performance regardless of age. Thus, while the functional significance and mechanistic underpinnings of age-related neural dedifferentiation remain to be fully elucidated, individual differences in neural differentiation appear to reflect both age-dependent and age-invariant factors. Future research should examine the factors driving individual differences in neural differentiation regardless of age. Additionally, longitudinal rather than cross-sectional designs using larger and more diverse samples are required to elucidate how neural differentiation is affected by aging and whether changes in neural differentiation predict cognitive change.

## Synthesis

Reviewing Editor: Morgan Barense, University of Toronto Decisions are customarily a result of the Reviewing Editor and the peer reviewers coming together and discussing their recommendations until a consensus is reached. When revisions are invited, a fact-based synthesis statement explaining their decision and outlining what is needed to prepare a revision will be listed below. Note: If this manuscript was transferred from JNeurosci and a decision was made to accept the manuscript without peer review, a brief statement to this effect will instead be what is listed below.

Thank you for submitting your paper to eNeuro. I’ve carefully reviewed your responses to your past reviewers. I’m convinced that you have thoroughly addressed all the concerns and I see no need to send it for further review. This is a well-executed and well-written study on an important topic, and will be a valuable contribution to the literature. I know that my lab will be citing it!

Best,

Morgan
